# Influence of On-Sight and Flash Climbing Styles on Advanced Climbers’ Route Completion for Bouldering

**DOI:** 10.3390/ijerph182312594

**Published:** 2021-11-29

**Authors:** Jesús Morenas, Vicente Luis del Campo, Sergio López-García, Lucía Flores

**Affiliations:** 1 Laboratory of Motor Control and Learning, Faculty of Sports Sciences, University of Extremadura, 10003 Cáceres, Spain; jesusmorenas@unex.es (J.M.); viluca@unex.es (V.L.d.C.); luciaffvv@gmail.com (L.F.); 2 Faculty of Education, Pontifical University of Salamanca, 37007 Salamanca, Spain; 3 Group on Physical Activity, Sports and Health (GIADES), Pontifical University of Salamanca, 37007 Salamanca, Spain

**Keywords:** route preview, perceptual-cognitive, climbing performance, on-sight climbing, flash climbing

## Abstract

Route previewing has been established as a critical parameter in indoor climbing performance, as it could determine the success or failure in ascending the route. We addressed the effect of different types of previews on output climbing performance. Twenty-one advanced climbers (7b and 7c+ climbing grade) were required to complete 18 routes, rated at 6c, according to the French Rating Scale of Difficulty. Each climber previewed the route under three conditions: “No-previewing”, “video-model previewing”, and “real-model previewing”. Output climbing performance was assessed in terms of route completion. The results showed differences on output climbing performance between types of preview. Specifically, the climbers achieved more successful attempts at climbing to the “Top” of the wall when inspecting the route with the “real-model previewing” condition, compared to the other conditions of preview. On the contrary, the climbers displayed more failed attempts in climbing the route with the “on-sight” condition, compared to the “flash” styles (“video-model” and “real-model”). The preview of the route, including performance of a real/video-projected model manipulating climbing holds, seems to increase the opportunities to climb the boulder successfully, attuning climbers to information specifying ascending actions. Climbing coaches should reinforce the design of representative training, using flash styles, to promote movement solutions for route completion.

## 1. Introduction

Sport climbing has gained popularity in society and wider practice among the population during recent decades, achieving recent inclusion in the Tokyo 2020 Olympic program [1]. In indoor climbing competitions, the final score is determined by several factors, depending on the climbing discipline. To exemplify, in lead, climbing ability is determined by the handhold use on the climbing wall, and the total time spent to complete the route. In boulder, climbing ability is determined by the number of routes (commonly known as *problems* or *boulders*) completed and the total number of attempts. Finally, in speed, climbing time is the main factor in performance [2].

Recent investigations have focused on kinematic, anthropometric, nutritional, psychological, and physiological factors in climbing performance [3,4,5,6,7,8]. For example, expert climbers showed higher levels of planning performance and memorizing of climbing movements, compared to novice climbers. These cognitive skills could underlie the solution to “problems” while ascending the route [7]. Some studies have shown that psychological variables could be crucial in achieving high-performance levels in sport climbing, such as problem-solving ability and movement sequence recall [9]. Similarly, the type of route previewing strategy would determine the climbers’ gaze behaviour (e.g., number of visual patterns and fixation durations) [10]. 

There are three climbing styles needed to successfully climb to the top of a route: On-sight, where the climber completes a route without augmented information provided by the coach or prior knowledge of the new route; Flash, where the climber completes a climb on the first attempt after receiving specific information about the route; and *Redpoint*, where the climber completes a route without falling after making previous unsuccessful attempts, rappelling down the route or interceding with a top rope [6,11]. The on-sight style is based on a standard protocol used in both indoor lead climbing competitions, where all competitors are only allowed to make a visual inspection of the route before ascending it [2]. In boulder competition the climber may reach the top of the climbing wall after several attempts but could gain an advantage if the top is reached on the first attempt. 

Regarding route previewing, this factor determined form performance (number and duration of moves and stops), but not output performance (route completion) of a sample of climbers with different skill levels. Specifically, the climbers made fewer and shorter stops during their ascent following a preview of the route. However, no benefits were found in finishing the ascend when visual inspection was offered [12]. Route previewing may be considered as a form of “non-physical practice” because the pre-ascent climbing route would include a visual inspection of the route from the ground. However, climbers could use the time visualizing the route to evaluate structural properties of the boulder wall (e.g., the shape or size of the holds for low-skilled climbers) or their functional properties (e.g., what actions would be needed to grasp the holds for high-skilled climbers), for example, simulating specific movements to climb the wall with physical gestures [13].

Fingertip strength was another variable influencing climbers’ visuo-motor behaviors on new routes [14]. As a result, stronger climbers demonstrated a more complex visual and motor behaviour climbing the routes because their opportunities to act remained independently of the route difficulty. Contrary, the weaker climbers displayed less complex behaviour because of their lack of ability to perceive and act in this specific environment. However, errors in previewing the route have been identified as a major reason for falling during climbing [15]. In this vein, the role of route previewing may also be decisive when the on-sight climbing style was performed [16]. 

Route previewing may help climbers perceive affordances offered by the surface of the boulder wall [17], collecting functional information about reachable, graspable, and usable holds in order to assemble movements and to find the best route [8]. Different visual patterns could emerged during the preview of a route: ascending, fragmentary, or zigzagging strategies, and sequence-of-blocks [18]. According to these authors, this last perceptual strategy was the most commonly used for intermediate and advanced routes because it was connected with tactical training. Another effect of perceiving opportunities for actions during route previewing would be the enrichment of the recall skill for those holds associated with the action performance [15]. For example, expert climbers were more accurate in perceiving specific affordances on the climbing wall, underlined by a superior ability to recall visual cues (holds) and motor sequences for routes [19].

These previous studies reveal effects of route previewing in climbing performance. However, it is unknown whether some types of instruction method provided by climbing coaches (i.e., model learning vs. the video technology) would be more appropriate during the preview than the on-sight style to improve the rates of route completion. The direct observation of a real model and video feedback technology is considered as augmented information [20], helping climbers to stabilize the coupled perception and action processes within the specific training environment [21]. In this vein, these instruction methods would facilitate climbers’ processes when searching for functional task solutions [22], or for stable movement coordination [23].

In this study we compare in a novel way the effect of two different flash climbing styes (“video-model” and “real-model”) and the on-sight climbing style on climbers’ output performance. We hypothesized (first hypothesis) that the climbers would achieve more success climbing the wall when perceiving a route via an instruction method (i.e., using the “video-model” or the “real-model”) compared to the on-sight style. Thus, we hypothesized (second hypothesis) that the climbers would achieve better route completion with a “real-model” condition, compared to the flash style (“video-model”) and the on-sight style. 

We considered that the lack of augmented visual information for the on-sight condition would conceal from climbers those affordances needed for route completion, available from the observation of real and/or video-projected specific climbing movements performed by an expert climbing model. Additionally, we reasoned that the “real-model” condition would better attune climbers to the information that specified those movement patterns needed to climb the wall, compared to the “video-model” condition. In this vein, we argue that the direct demonstrations performed by a real model on the climbing wall would better guide climbers’ self-regulation of actions when climbing the boulder wall in practice [20]. 

## 2. Materials and Methods

### 2.1. Ethical Approval

Participants voluntarily took part in the study and gave written informed consent to participate in this investigation, which was carried out according to the guidelines of the University’s Ethics Committee and the Declaration of Helsinki. Ethical approval was provided by the Bioethics and Biosafety Committee of the University of Extremadura (approval number: 33/2018). All participants were informed about the general objectives but were naïve to the specific hypothesis of the research. 

### 2.2. Participants

Twenty-one advanced climbers (7 female and 14 male), with a mean age of 21 years (±ewere recruited for this study. The anthropometric indices of climbers’ bodies (in mean vales) were: 171 cm for height (±8.2), 71 kg for body mass (±10.9), 171 cm for arm span (±11.8), and 0.99 for ape index (or arm span to height ratio) (±0.03).

As criteria of participation, the climbers had high ability in boulder climbing, ranging from 7b to 7c+ (level 3 on the French Rating Scale of Difficulty: F-RSD) [24,25], and a minimum of 3 years of training experience. Current on-sight ability (last two months) was requested to avoid initial differences in climbing performance level. All necessary safety measures were taken to prevent accidents and/or injuries. This number of participants was based on a power analysis for Wilcoxon-Mann-Whitney tests with two tails, α = 0.05, effect size = 0.8, 1 − β = 0.8, required sample size = 15 [26].

### 2.3. Climbing Routes

18 boulder routes of the same difficulty level, all identifiable by color, were set on an indoor climbing wall by one professional route setter certified by the Spanish Climbing Federation. All routes had the same number of holds (six), including one starting and one top handhold. The routes were set at the same difficulty level, rated at 6c, and considered as intermediate/advanced level routes on the F-RSD. Climbers had no information about the grade of the route.

Within this difficulty level, some routes were designed with a more pronounced technical, physical or perceptual component to provide a more representative climbing environment with which to evaluate the climbers’ output performance, preventing bias from possible differences between climbers in their technical abilities, physical capacities and/or perceptual skills. Specifically, there were six routes marked with a high physical component to ascend the climbing wall (i.e., routes that required great effort using the fingers or upper limb strength); six routes with a high technical component (i.e., routes that required a great demand of the motor repertoire, for instance, heel or toe hook, twisting, crossover or Gaston); and six “mixed routes” (i.e., routes that required a combination of physical and technical components, but also perceptual skills). 

### 2.4. Procedures

**Figure 1:**
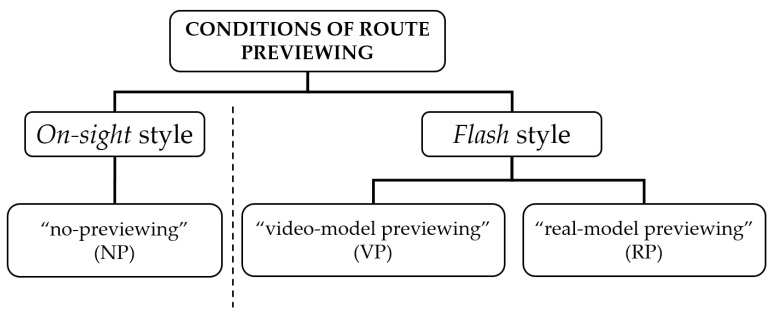
Conditions established of route previewing.

Firstly, the climbers were invited to warm up in the same way that they normally do when attempting climbs (e.g., performing the routes with their own equipment such as climbing shoes or chalk bag). The participants had only one attempt at each route, resting for 5 min between them for total recovery [14]. They were instructed not to perform previous attempts or touch the handholds. Three conditions of route previewing were established (Figure 1). 

Specifically, these conditions of initial visual inspection of the climbing wall included the on-sight style or the “no-previewing” condition (NP) (i.e., the participants only observed the climbing wall), and two flash styles: the “video-model previewing” condition (VP) (i.e., the participants watched a video recording that included an expert climber performing the route on a climbing boulder), and the “real-model previewing” condition (RP) (i.e., the direct observation of the same expert climbing the route on the same boulder as in the VP condition). Climbers were allowed to visually inspect the route for a limited time (4 min) with the “real model” and “video model” conditions, just before climbing the wall, as is usual in the competitions of the International Federation of Sport Climbing. Finally, all participants strived to climb the 18 routes in the same order as determined by the research team. 

### 2.5. Measurements

Measurement of age, height, body mass, arm span, and ape index (ratio of arm span to body height) were carried out to specify anthropometric indices of climbers’ bodies, using an electronic stadiometer scale (Seca 769 Digital Medical Scales with BMI, Hamburg, Germany) [27]. A camera (SJCAM sj5000x elite, Shenzhen, China) was used to record climbers’ performances. This action camera, 4 m away from the boulder, and provided with full HD resolution, recorded climbers’ attempts at ascending the route. 

The output climbing performance was scored in terms of route completion (“Yes”: Successful attempts occurred when participants climbed the top of the wall, and “No”: Failed attempts occurred when participants did not climb to the top of the wall). Additionally, this output performance was also assessed similarly to the competition rules: “Top” (when the climber reached the upper zone of the climbing wall, grasping the highest handhold), “Zone” (when the climber reached the middle zone of the wall, grasping the zone handhold), and “*Fail*” (when the climber did not reach either the “Zone” or the “Top”).

### 2.6. Statistical Analysis

The independent variable was the Type of preview (Level 1: A preview of the climbing wall but without information from recordings or real performances of the expert climbing the boulder wall, Level 2: A video preview of an expert climbing the boulder wall, Level 3: A preview of the same real expert climbing the boulder wall).

Shapiro-Wilks and Levene analyses confirmed that the data for output climbing performance did not display a normal distribution, and therefore nonparametric tests were used in this study. Firstly, a Chi-Squared test was used to determine differences in percentages of Fails, Zones, and Tops between types of preview. Subsequently, the Friedman test was carried out to address differences between mean ranges for the three previewing conditions. In case of reporting differences, the Wilcoxon test was used to determine pairwise comparisons in these types of preview.

The effect sizes (*ES*) were calculated for significant differences between pairs of previews, providing a better interpretation of the results. The mean difference between these pairwise comparisons divided by their pooled standard deviations was used to estimate the magnitude of *ES*. Specifically, three categories of Cohen [28] were used to interpret ES (small: *d* = 0.20; medium: *d* = 0.50; and large: *d* = 0.80). The odds ratios (ORs) were also used as an additional effect size to compare the relative likelihood for a specific outcome between two or more groups [29]. The odds ratio was calculated by the formula: AD/BC (A = group 1/outcome 1; B = group 1/outcome 2; C = group 2/outcome 1; D = group 2/outcome 2 [30]. In our study, the letters A/B/C/D represent observed cell frequencies when compared to two types of preview (i.e., NP vs. VP, NP vs. RP, VP vs. RP) and two possible outcomes for route completion (“Yes” = The climbers reach the top of the climbing wall; “No” = The climbers did not reach the top of the climbing wall).

The confidence intervals for effect sizes (*CI*) were also calculated to provide a practical value of the study in real-world terms [31]. CIs for effect sizes were calculated with the formula: 95% CI = ES − 1.96 *se* to ES + 1.96 *se* [32]. Finally, we calculated the number needed to treat (NNT) as another indicator to clarify the practical value of effect sizes. The NNT score is the number of participants who must be treated to give one more success/one more minor failure as one outcome of an intervention. The NNT effect size indicator is used when there are dichotomous outcome variables [29]. To calculate this indicator, we consider again the output climbing performance with two possible outcomes, as previously assigned to the ORs. The score from this calculation is discussed as risk difference (RD). One formula for calculating the NNT, using the RD score, is 1/RD. In this formula, the result of 1 is the best NNT score indicating that the treatment is perfect (e.g., all climbers in one previewing condition have improved, whereas no climber in another previewing group had) [31]. An alpha level of <0.05 was set for all analyses.

## 3. Results

Firstly, the nonparametric tests revealed that the data of the dependent variable did not display a normal distribution for the route previewing. Specifically, the results found in the Shapiro-Wilks analysis were RP (0.683; *p* < 0.001), NP (0.767; *p* < 0.001), NP (0.784; *p* < 0.001), and *W* = 19.94; *p* < 0.001 for the Levene analysis.

Table 1 shows the number and percentages of climbing attempts resulted as Fails, Zones and Tops achieved by the sample of climbers when observing the boulder according to the different types of previewing condition, to highlight that the RP enhanced the climbers’ output performance because it was the only condition with percentages of Tops above 50%. The results revealed significant differences in percentages of Fails, Zones, and Tops between the three types of preview (*X*^2^ = 56.29; *p* < 0.001). 

There were differences between mean ranges of these different route previews (*X*^2^ = 13.81; *p* < 0.01). The pairwise comparisons displayed differences between RP vs. NP conditions (*Z* = −3.41; *p* < 0.01), and between RP vs. VP conditions (*Z* = −3.40; *p* < 0.01). No significant difference was found for the comparison between the VP and NP conditions (*Z* = −1.77; *p* = 0.07). Specifically, the values of mean ranges were 2.60 (RP), 1.86 (VP), and 1.55 (NP). The effect size was large for the comparison between RP and NP conditions (see Table 2), and small for the rest of pairwise comparisons (RP vs. VP, and NP vs. VP). 

Table 3 shows that the exposure to the RP increased by more than seven times the probability of climbing to the top of the wall compared to the NP, and more than two times compared to the VP. This VP increased by more than three times the probability of climbing to the top of the wall compared to the NP. Specifically, the lower and upper 95% CI for ORs were 4.41 and 11.22 when compared the RP and NP conditions; 1.31 and 2.97 comparing the RP and VP conditions; and 2.10 and 5.47 for the comparison between the VP and NP conditions. 

Table 4 shows that the calculated NNT score indicated that approximately one climber out of three had a successful attempt at climbing the “Top” of the wall when they had observed the boulder with the RP condition compared to the NP one.

Also note that this NNT score indicated that approximately one climber out of six, and one climber out of five climbed to the “Top” of the wall having observed the boulder with the RP condition compared to the VP one, and with VP condition compared to the NP, respectively.

## 4. Discussion

This study aimed to investigate the impact of route previewing with an on-sight style and different flash styles that included different instruction methods provided by the research team (i.e., model learning with a direct visual demonstration vs. video technology), on advanced climbers’ performance (in terms of route completion) for a bouldering climbing style. Firstly, it is highlighted that the participants obtained better output climbing performance when they received an instruction method. As a result, the climbers achieved higher percentages of successful attempts at climbing to the top of the wall during the flash styles (i.e., during the “real-previewing” and the “video-previewing” conditions), compared to the on-sight style (i.e., during the “no-previewing” condition). This positive influence of route previewing on climbing performance has been previously found by Sanchez et al. [12], and Seifert et al. [10]. 

To highlight that the flash climbing styles, as instruction methods of learning in climbing, caused a true impact on the advanced climbers’ output performance, the effect sizes were large (when comparing the “real-model” to the “no previewing” conditions), and nearby to medium (comparing the “video-model” to the “no previewing” conditions). In addition, the confidence intervals for ESs did not include zero or a negative number, and therefore there was a 95% likelihood that a true population effect occurred between the lower and the upper scores of these statistics (i.e., to report that a finding does exist in the real world). According to these statistics, the first hypothesis of the study is accepted.

The NNT score also indicated that approximately one climber out of five had a beneficial outcome (i.e., to climb to the “Top” of the boulder) due to the “video previewing” condition, compared to “no previewing” one. These findings reinforce the use of video modeling technology as an effective strategy to improve skill performance following this video intervention in a wall climbing task [15]. These authors found that inexperienced climbers improved their climbing performance after watching videos of an expert climber performing the same route. This preview compensated for their lack of ability in reading the route to climb to the top of the wall.

A substantive rationale for understanding climbers’ output performance in this study would be the contribution of action observation in action execution [33]. From this approach, the observation of an object would activate a motor simulation of the actions needed to achieve the desired goal [13]. For example, the perception of climbing holds would evoke the corresponding reaching and/or grasping postures [34]. However, we consider that the differences between route previews would be better explained by the affordance-matching hypothesis. According to this view, the object knowledge (i.e., what an object is for and how it is used) would have a prominent role for action understanding [35]. To exemplify, the observed grasping actions performed by an expert model interacting with the climbing holds would act to a corresponding action in the climber’s motor repertoire [36]. 

To our best knowledge, we reasoned that the direct observation of an expert model manipulating climbing holds when ascended the boulder, but also the preview of video-recordings of this model performing climbing actions, may help climbers’ action prediction; for instance, guiding climbers’ perception towards action-relevant objects (i.e., how to use the climbing holds for grasping actions). In this vein, the observed actions performed by the “real-model” and/or “video-model” would predict forthcoming actions of the climbers due to a more efficient identification and interaction with the climbing holds (i.e., manipulation knowledge or knowledge of how the grasping actions should be performed to ascend the route). Nevertheless, the only inspection of the climbing holds available on the boulder would provide a scarce interpretation of what holds are for but not of how they should be used (i.e., function knowledge or knowledge of what climbing actions should be performed to climb the wall) [35]. As a result, climbers showed higher output performance during the flash styles, compared to the on-sight style, because they observed augmented visual information during the preview, via inspection of the climbing holds, but also via inspection of climbers’ reaching and grasping actions in performing movement solutions to climb the wall.

The preview of the route using a real expert climber improved the ability of participants when ascending the boulder, compared to the other previewing conditions. For example, the NNT score indicated that: (i) approximately one climber out of three climbed to the “Top” of the boulder when previewing this climbing wall with the “real-model” condition compared to “no previewing”, (ii) approximately one climber out of six ascended the route when observing the “real-model” condition compared to the “video-model”. The “real model” previewing also reported a tendency toward more successful attempts at climbing the wall than the “video-model”, but not at a significant level. Therefore, in light of these findings, the second hypothesis would be partially accepted. 

We argue that the direct observation of specific climbing actions performed by an expert model on the boulder enhanced climbers’ ability to climb this wall via perceptual sensitivity, as this type of route previewing offered opportunities to inspect relations between the model’s body segments. In particular, the climbers observed performances close to the boulder, ensuring a natural depth perception due to a wider viewing angle [37]. Most importantly, these real performances made by the expert model ensured a higher representativeness of the climbing performance environment [38], helping climbers towards a deeper understanding of the expert model’s reaching and grasping actions, compared to the video recordings of this model. On the contrary, the video technology offered a biased display of the expert model–boulder interaction because the information about the climbing actions involved in their attempts to climb the wall was partially masked by the lack of a three-dimensional presence (i.e., there was a loss of information about relationships between body segments in terms of rotations about one or more axes of the joints connecting them) [39].

The direct observation of an expert climber could create increased opportunities for affordance perception on the boulder wall, enhancing climbers’ output performance (e.g., guiding climbers’ attention toward the informational variables affordable in the expert model–boulder relationship for climbing the wall) [40]. In this vein, the preview of the route with a “real-model” better specified the environmental properties of the boulder, offering invitations to act. Altogether, the preview of a real climber performing specific climbing movements created a better comprehension of kinematic information embedded in the boulder, entailing climbers’ ability to perceive what the climbing wall offers them relative to their abilities [41]. Therefore, the real model would reinforce the relationship between affordances and visual information contained on the climbing wall, providing a landscape of opportunities for action [42]. As a result, climbers would achieve more functional and adaptive movements for route completion.

Collectively, to posit that the flash climbing styles caused a true impact on the advanced climbers’ route completion, compared to *the on-sight* style, when comparing both instruction methods, no significant differences were found, although there is a tendency towards better output performance for the “real-model” condition compared to the “video model” one. It seems that the preview of an expert model performing specific climbing movements facilitates climbers’ learning from that functional visual information available on the boulder relevant for ascending this type of climbing wall. In doing so, the affordance approach but more specifically the affordance-matching hypothesis seems to be a plausible niche to explain differences in advanced climbers’ route completion with manipulations of route previewing styles.

## 5. Conclusions

The output climbing performance was sensitive to changes in route previewing. The flash climbing styles increased the opportunities to ascend the route, achieving higher percentages of route completion, compared to the climbing on-sight style. Therefore, the use of an instruction method during the training sessions (e.g., the model learning and/or the video technology) improved the participants’ output climbing performance. Additionally, the direct demonstrations performed by an expert resulted in the most effective method to enhance climbers’ route completion. However, the differences in terms of output performance were not significant when comparing the two flash climbing styles, resulting in a small effect size.

## 6. Strengths and Limitations

This research study had two main advantages. The first was to provide new insights about the importance of route previewing on climbers’ output performance because this ability to visually inspect the route before ascending has been a key parameter that predicted indoor climbing performance [10]. Second, the results of this study revealed that the route completion was affected by the type of route previewing, and therefore these findings should be treated with attention by the climbing coaches. For example, they should handle the development of training with flash climbing styles to afford climbers proper tactical decisions and manageable action plans in climbing the boulder following the presentation of the route previewing with specific reaching and grasping actions performed by other climbers. 

On the contrary, this research had two main concerns. First, this study only investigated the output performance of the climbers (i.e., the route completion) but not their form climbing performance (e.g., the number and duration of movements and stops ascending the route) [10]. This analysis of the “form” could reveal what type of exploratory and performatory movements were made by the climbers during their attempts to ascend the route [43]. Second, no gaze information for climbers was collected. Eye tracking technology could discover whether the different types of route previewing would impact on climbers’ visual behaviors and how possible differentiating gaze patterns could influence the ascent of the boulder.

In future studies, it would be interesting to address whether this tendency towards output performance with the “real model” condition, compared to the “video model”, would achieve significant levels with larger samples of climbers. Virtual reality to create three-dimensional environments using computer graphics or 360° video imagery would be another prominent technology to test their influence on optimal climbing performance. In this vein, virtual reality could offer opportunities to simulate specific climbing environments in which climbers could have opportunity to perform particular grasping actions in an enriched boulder composed of many types of holds (e.g., slopers, jugs, edges, pockets, pinches) with different structural (e.g., orientation and shape) and functional (e.g., grasping and reaching opportunities for each hold) features.

Future studies of climbing should investigate what model of motor learning (explicit vs. implicit) would be more appropriate to enhance both the “form” and “output” of advanced climbersperformance in bouldering when using video feedback. For example, would the climbers improve, for instance, route finding, planning, and problem-solving when they received instructions from their coaches by guiding visual attention to the relevant information of the boulder and/or other climbers’ movements available in the video recordings, or with a strategy of self-controlled video feedback in which the climbers themselves could scan possible solutions for climbing the wall during the observations of these recordings? This last option could be a suitable strategy to promote self-regulatory processes of action in climbers when ascending boulders.

## 7. Practical Perspective

According to the results of this experiment, we would recommend to climbing coaches the use of instruction methods including external visual information (e.g., model learning and video feedback) to optimize the design of training for advanced climbers. This augmented information available from the observation of real and/or video recordings of other climbers’ performances could be useful; for instance, to enhance a (re)organization of climbers’ perception–action couplings (during the skill adaptability training stage) and/or to acquire self-regulatory technical-tactical skills in competition (during the performance training state) [22]. 

Along these lines, we would encourage climbing coaches to assemble video footages to their climbers, introducing video recordings of the best climbing counterparts’ performances for bouldering during training regimes. Additionally, the use of additional viewpoints such as the side view for the “video model” condition could help climbers towards a better assessment of the tri-dimensionality of motion. Thus, it would be appropriate to train with other teammates of similar climbing skill level to direct climbers to different manipulations of climbing holds made by other climbers. These previews would enhance object knowledge beforehand for climbing the rout.

Finally, flash climbing styles could also be used during the climbers’ injury periods to direct them to specific information that directly relates to the perceived property of action (e.g., the reaching and grasping actions performed by the expert model on the climbing wall) and to re-calibrate climbers’ possibilities for action on the boulder (e.g., estimating how their boundaries of reaching-to-grasp change along this recovery time). 

## Figures and Tables

**Table 1:** Output performance in number and percentages of Fails, Zones, and Tops for the sample of climbers when observing the boulder with different types of preview.

			Attempts		
			Fails	Zones	Tops
Previewing	NP	Number of cases	23	80	23
		Percentage	18.25%	63.49%	18.25%
	VP	Number of cases	26	46	54
		Percentage	20.63%	36.50%	42.86%
	RP	Number of cases	6	43	77
		Percentage	4.76%	34.12%	61.11%

Legend: NP = no previewing; VP = video previewing; RP = real previewing.

**Table 2:** Effect sizes (*d*) and confidence interval for effect size (CI) for the comparisons between types of previewing condition.

Previewing				Effect Size	Confidence Interval for Effect Size (CI)	
	Mean	*n*	SD		Lower	Upper
RP NP	1.56	126	0.58	0.95	0.69	1.21
	1	126	0.60			
RP VP	1.56	126	0.58	0.49	0.24	0.74
	1.22	126	0.78			
NP VP	1	126	0.60	0.32	0.07	0.56
	1.22	126	0.78			

Legend: NP = no previewing; VP = video previewing; RP = real previewing.

**Table 3:** Odds ratios (OR) using the number of trials with success (“Yes” = Top) and not success (“No” = Zone or Fail) when comparing the different types of preview performed by the sample of climbers.

		Yes	No
Previewing	NP	23.00	103.00
	VP	54.00	72.00
	RP	77.00	49.00
	OR comparing RP vs. NP		7.04
	OR comparing RP vs. VP		2.10
	OR comparing VP vs. NP		3.36

Legend: NP = no previewing; VP = video previewing; RP = real previewing.

**Table 4:** Number needed to treat (NNT) for success (Yes = Top) and percentage of success (%) above the total cases (*n* = 126) when compared to the no previewing condition (NP) against video (VP) and real (RP) previewing conditions for the sample of climbers.

		Yes	%
Previewing	NP	23.00	18.25
	VP	54.00	42.86
	RP	77.00	61.11
	Percentage difference	NNT	NNT*100
RP vs. NP	42.86	0.02	2.33
RP vs. VP	18.25	0.05	5.47
VP vs. NP	24.60	0.04	4.06

Legend: NP = no previewing; VP = video previewing; RP = real previewing.

## Data Availability

The data presented in this study are available on request from the corresponding author. The data are not publicly available due to due to privacy.
